# G9a orchestrates PCL3 and KDM7A to promote histone H3K27 methylation

**DOI:** 10.1038/srep18709

**Published:** 2015-12-21

**Authors:** Mei-Ren Pan, Ming-Chuan Hsu, Li-Tzong Chen, Wen-Chun Hung

**Affiliations:** 1 Graduate Institute of Clinical Medicine, College of Medicine, Kaohsiung Medical University, Kaohsiung 804, Taiwan; 2 National Institute of Cancer Research, National Health Research Institutes, Tainan 704, Taiwan; 3 Division of Hematology/Oncology, Department of Internal Medicine, National Cheng Kung University Hospital, College of Medicine, National Cheng Kung University, Tainan 704, Taiwan; 4 Institute of Basic Medicine, College of Medicine, National Cheng Kung University, Tainan 704, Taiwan

## Abstract

Methylation of histone H3-lysine 9 (H3K9) and H3K27 by the methyltransferase G9a and polycomb repressive complex 2 (PRC2) inhibits transcription of target genes. A crosstalk between G9a and PRC2 via direct physical interaction has been shown recently. Here, we demonstrate an alternative mechanism by which G9a promotes H3K27 methylation. Overexpression of G9a increases both H3K9 and H3K27 methylation, reduces E-cadherin expression, and induces epithelial-mesenchymal transition in PANC-1 pancreatic cancer cells. Conversely, the depletion of G9a or ectopic expression of methyltransferase-dead G9a in G9a-overexpressing gemcitabine-resistant PANC-1-R cells exhibits opposite effects. G9a promotes H3K27 methylation of the E-cadherin promoter by upregulating PCL3 to increase PRC2 promoter recruitment and by downregulating the H3K27 demethylase KDM7A to silence E-cadherin gene. The depletion of PCL3 or overexpression of KDM7A elevated expression of E-cadherin in PANC-1-R cells while ectopic expression of PCL3 or knockdown of KDM7A downregulated E-cadherin in PANC-1 cells. Collectively, we provide evidence that G9a orchestrates the dynamic balance within histone-modifying enzymes to regulate H3K27 methylation and gene expression.

Transcriptional activity of mammalian genes is controlled by epigenetic regulation including DNA methylation and histone modification. DNA methylation at the CpG sites of gene promoters usually induces transcription inhibition^1^,^2^. Conversely, the effect of histone modification on gene expression is complex due to histone proteins can undergo many post-translational modifications (PTM) including phosphorylation, acetylation, methylation, sumoylation, ubiquitination, etc. In addition, the effect of PTM on transcriptional activity is dependent on the residues modified, the location of the modifications happened and the degree of modification^3^,^4^,^5^. For example, the lysine residues of histone H3 can be mono-, di-, or tri-methyled (m1, m2, and m3) by different histone methyltransferase (KMTs). Methylation on lysine 4 (H3K4) or 36 (H3K36) is usually associated with transcriptional activation while H3K9 and H3K27 methylation are frequently linked with gene silencing and are hallmarks of chromatin condensation^5^,^6^,^7^,^8^.

Recent studies demonstrate that the crosstalk between different histone modifications orchestrated by histone modifying enzymes is critical to fine-tune gene transcription to fit the physiological demands and to mediate the pathological changes of cells. H3K27 demethylase KDM6A up-regulated gene expression programs associated with growth and invasion of breast cancer cells^9^. Interestingly, a cohort of the KDM6A-regulated genes is also targets of the H3K4 methyltransferase MLL4. The authors showed that KDM6A directly interacts with the c-terminal region of MLL4 and the coordinated regulation of gene transcription by MLL4 and KDM6A promotes proliferation and invasion of breast cancer cells. Another elegant study revealed a novel mechanism of the coordinated H3K4 methylation/H3K9 demethylation in the control of gene expression^10^. *In vivo*, these two histone markers are mutually exclusive. Immunoprecipitation and mass spectrometry analysis demonstrated that H3K9 trimethyl demethylase JMJD2B is a component of the mixed-lineage leukemia (MLL) 2 complex, a H3K4-specific methyltransferase. Functional characterization of the JMJD2/MLL2 complex showed that this complex could be co-purified with estrogen receptor α and is important for estrogen receptor α-mediated transcription. The interplay between two repression complexes to induce gene silencing has recently shown by the study of the H3K9 methyltransferase G9a and the H3K27 methyltransferase complex PRC2^11^. G9a is a key methyltransferase to introduce mono- and di-methylation of H3K9. Although G9a has been shown to methylate H3K27 *in vitro*, whether G9a could directly methylate H3K27 *in vivo* is still controversial. Mozzetta *et al.*^11^ demonstrated that G9a physically interacts with PRC2 and modulates PRC2 genomic recruitment to specific target genes to increase H3K27 trimethylation at genomic loci. These results provide a molecular basis by which G9a controls H3K27 methylation via direct coupling with PRC2. Here, we revealed a novel mechanism by which G9a coordinates the expression of various histone modifying enzymes to promote H3K27 methylation and to inhibit gene transcription.

## Results

### G9a induces H3K9 and H3K27 methylation and downregulates E-cadherin in pancreatic cancer cells

**Figure 1:**
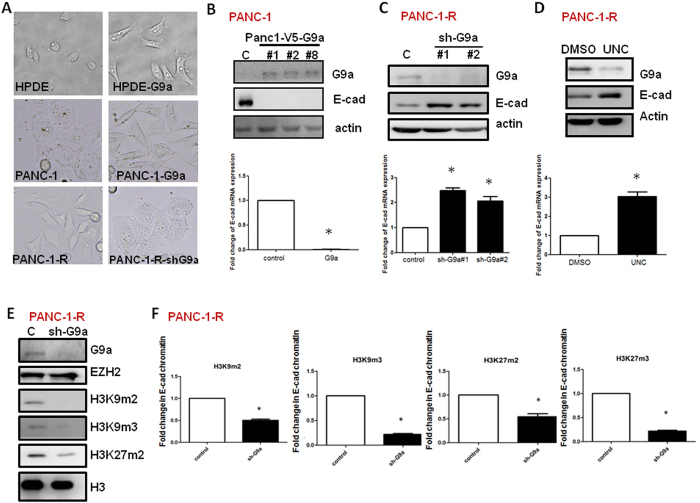
G9a induces H3K9 and H3K27 methylation and downregulates E-cadherin in pancreatic cancer cells. (**A**) Morphology of control, G9a-overexpressing (HPDE-G9a, PANC-1-G9a) cells or G9a-depleted (PANC-1-R-shG9a) cells. (**B**) Ectopic expression of G9a inhibited E-cadherin expression. The protein levels of G9a and E-cadherin in control and G9a-overexpressing PANC-1 cells were verified by Western blot analysis (top panel) and E-cadherin mRNA level was determined by RT-qPCR analysis. *p < 0.05. (**C**) PANC-1-R cells transfected with control or various G9a shRNAs were harvested for the analysis of E-cadheirn protein (top panel) and mRNA (low panel). *p < 0.05. (**D**) PANC-1-R cells were treated with vehicle (DMSO) or UNC0638 (500 nM) for 48 h and the expression of E-cadherin protein and mRNA was investigated. *p < 0.05. (**E**) PANC-1-R cells were infected with control or G9a shRNA-containing lentivirus. After 48 h, G9a levels and histone methylation status were investigated by Western blot analysis. (**F**) PANC-1-R cells were infected with control or G9a shRNA-containing lentivirus. ChIP-qPCR analysis was performed to investigate the status of H3K9m2, H3K9m3, H3K27m2, and H3K27m3 in the E-cadherin gene promoter.

We had previously established a gemcitabine-resistant cell line (PANC-1-R) from the parental human PANC-1 pancreatic cancer cells^12^. Although PANC-1-R cells are highly resistant to GEM and exhibit high invasive characteristic (as shown below), the proliferation rate of these two cell lines is similar suggesting the phenotypic alterations are not related to their growth activity (Supplementary Fig. S1). We found that expression of G9a was significantly up-regulated in PANC-1-R cells (Supplementary Fig. S2). Interestingly, the methylation of H3K9 and H3K27 was also increased in PANC-1-R cells (Supplementary Fig. S2). These two cell lines are of similar genetic background and provide a good model for functional study of G9a. Overexpression of G9a in human pancreatic ductal epithelial cells (HPDE) and PANC-1 cells changed morphology from epithelial type to mesenchymal (spindle-like) phenotype (Fig. 1A). Conversely, PANC-1-R cells exhibited mesenchymal phenotype and the depletion of G9a by shRNA induced epithelial morphology associated with reduced migration and invasion abilities (Fig. 1A and Supplementary Fig. S3A). A similar inhibition was found by expressing methyltransferase-dead G9a indicating G9a promotes epithelial-mesenchymal transition and invasiveness in a methyltransferase-dependent manner (Supplementary Fig. S3A). The proliferation rate of PANC-1-R cells transfected with control or G9a shRNA was not significantly changed suggesting that the reduction of migration and invasion was not caused by an indirect effect of cellular proliferation (Supplementary Fig. S3B). Overexpression of G9a in PANC-1 cells inhibited E-cadherin expression while knockdown of G9a in PANC-1-R cells up-regulated it (Fig. 1B,C). Treatment with the G9a inhibitor UNC0638 also increased E-cadherin expression in PANC-1-R cells (Fig. 1D). In addition to inhibiting activity, UNC0638 also reduced G9a protein level after long-term incubation suggesting the enzymatic activity of G9a may be required for its protein stability (Fig. 1D). Knockdown of G9a reduced global methylation of H3K9 and H3K27 simultaneously without significant effect on the EZH2 level (Fig. 1E). Tri- and di-methylation of H3K9 and H3K27 of E-cadherin promoter were all decreased by G9a depletion (Fig. 1F). Overexpression of enzyme-dead G9a also reduced these methylation markers in PANC-1-R cells (Supplementary Fig. S4). The regulation of H3K9 and H3K27 methylation by G9a is not cell line- or cancer type-specific because knockdown of G9a in human A549 lung cancer cells also showed similar results (Supplementary Fig. S5A) and treatment of UNC0638 attenuated H3K27 methylation in A549 and MDA-MB-231 cells (Supplementary Fig. S5B). These results suggested that G9a increases methylation of H3K9 and H3K27 on the gene promoter of E-cadherin to repress its expression.

### G9a up-regulates PCL3 expression to promote EZH2 recruitment and H3K27 methylation of E-cadherin promoter

**Figure 2:**
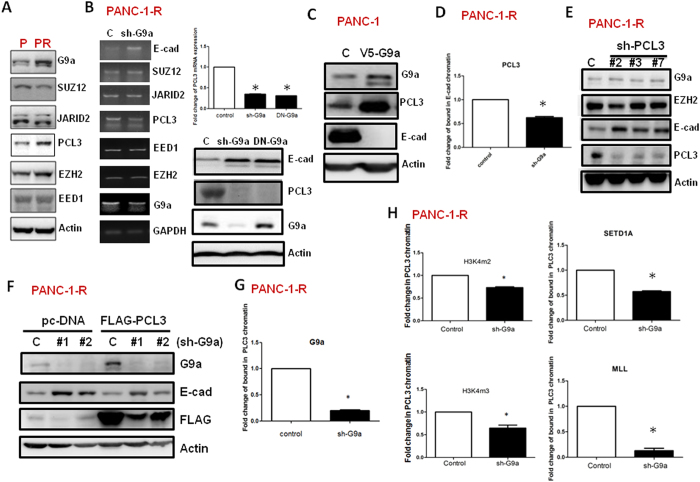
G9a up-regulates PCL3 expression to promote EZH2 recruitment and H3K27 methylation of E-cadherin promoter. (**A**) The protein levels of the PRC2 components of PANC-1 (P) and PANC-1-R (PR) cells were compared by Western blotting. (**B**) PANC-1-R cells were infected with control or G9a shRNA-containing lentivirus. RT-PCR analysis was performed to investigate the expression of the PRC2 complex components (left panel). RT-qPCR analysis was done to investigate the expression of PCL3 in PANC-1-R cells transfected with control or G9a shRNA or overexpressed methyltransferase-dead G9a (DN-G9a) (right-top panel). Protein levels of E-cadherin, PCL3 and G9a were studied by Western blot analysis (right-bottom panel). (**C**) Detection of G9a, PCL3 and E-cadherin expression in control or G9a-overexpressing PANC-1 cells by using Western blot analysis. (**D**) ChIP-qPCR analysis of the binding of PCL3 to E-cadherin promoter in control and G9a depleted cells. *p < 0.05. (**E**) PANC-1-R cells were infected with control or PCL3 shRNA lentivirus. After 48 h, expression of various target proteins was determined by Western blot analysis with indicated antibodies. (**F**) PANC-1-R cells were transfected with control or G9a shRNA in combination with FLAG-tagged PCL3. The protein levels of G9a, PCL3 and E-cadherin were studied by Western blot analysis with indicated antibodies. (**G**) ChIP-qPCR analysis was performed to investigate the binding of G9a to human PCL3 promoter in control or G9a-depleted PANC-1-R cells. *p < 0.05. (**H**) The binding of SETD1A and MLL to human PCL3 promoter and the methylation status of PCL3 promoter were studied by ChIP-qPCR in control or G9a-depleted PANC-1-R cells. *p < 0.05.

We tested the possibility that G9a regulates the expression of the PRC2 complex components. Among the core components and accessory proteins, only the expression of PCL3 was significantly increased in PANC-1-R cells (Fig. 2A). In addition, the depletion of G9a only reduced PCL3 expression in PANC-1-R cells (Fig. 2B). Knockdown of G9a or ectopic expression of methyltransferase-dead G9a decreased PCL3 mRNA by 60% (Fig. 2B). In addition, PCL3 protein levels were more significantly reduced in these cells (Fig. 2B). PCL3 is a Tudor domain-containing protein which plays an important role in the recruitment of the PRC2 complex to CpG islands^13^. Overexpression of G9a in PANC-1 cells significantly up-regulated PCL3 expression (Fig. 2C). Chromatin immunoprecipitation-quantitative polymerase chain reaction (ChIP-qPCR) assay demonstrated that G9a depletion reduced the binding of PCL3 to E-cadherin promoter by 40–50% (Fig. 2D). Similarly, UNC0638 also induced a 50% of reduction of PCL3 promoter binding (Supplementary Fig. S6). Knockdown of PCL3 in PANC-1-R cells increased E-cadherin expression without affecting G9a indicating PCL3 is a downstream effector of G9a (Fig. 2E). In addition, ectopic expression of PCL3 reduced the up-regulation of E-cadherin induced by G9a depletion (Fig. 2F). We found that G9a directly bound to the PCL3 gene promoter and the binding was significantly reduced by G9a depletion (Fig. 2G). UNC0638 also decreased the expression of PCL3 and the binding of G9a to PCL3 promoter (Supplementary Fig. S7). The protein levels of other components of the PRC2 complex was not significantly affected (Supplementary Fig. 8). Moreover, inhibition of G9a reduced the recruitment of MLL and SETD1A, two histone methyltransferase complexes, to PCL3 promoter and the di- and tri-methylation of H3K4 indicating attenuation of gene activation (Fig. 2H). Collectively, these data suggested that G9a directly binds to PCL3 promoter to activate its transcription and the upregulation of PCL3 increases the recruitment of PRC2 complex to E-cadherin promoter to downregulate gene expression.

### Histone demethylase KDM7A is important for G9a-mediated silencing of E-cadherin

**Figure 3:**
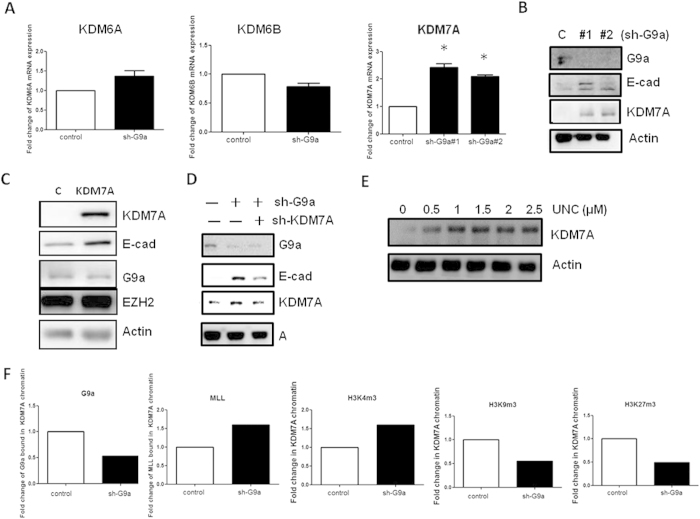
Histone demethylase KDM7A is important for G9a-mediated silencing of E-cadherin. (**A**) Expressions of three H3K27 demethylases KDM6A, KDM6B and KDM7A in control or G9a-depleted PANC-1-R cells were determined by RT-qPCR analysis. *p < 0.05. (**B**) The protein levels of G9a, E-cadherin and KDM7A in PANC-1-R cells transfected with control or various G9a shRNAs was investigated by Western blot analysis. Actin was served as a loading control. (**C**) The protein levels of KDM7A, E-cadherin, G9a and EZH2 in PANC-1-R cells transfected with control or KDM7A expression vectors were investigated by Western blot analysis. (**D**) PANC-1-R cells received different combined transfection of G9a or KDM7A shRNAs were harvested for Western blot analysis to determine the protein levels of G9a, E-cadherin and KDM7A. (**E**) PANC-1-R cells were treated with different doses of UNC0638 for 48 h and KDM7A protein level was studied. (**F**) The binding of G9a and MLL to human KDM7A promoter and the methylation status of KDM7A promoter were investigated by ChIP-qPCR. Results are averaged data of two independent experiments.

Because ectopic expression of PCL3 could not fully abolish the upregulation of E-cadherin induced by G9a depletion, we hypothesized the involvement of other G9a effectors. Three lysine demethylases including KDM7A, KDM6A and KDM6B have been identified to induce demethylation of H3K27^14^,^15^,^16^. As shown in Fig. 3A, the depletion of G9a significantly elevated the transcripts of KDM7A but not that of KDM6A and 6B. Increase of KDM7A protein was also evidenced in G9a-depleted clones (Fig. 3B). Overexpression of KDM7A increased E-cadherin without affecting G9a (Fig. 3C) and knockdown of KDM7A attenuated the increase of E-cadherin induced by G9a depletion (Fig. 3D). UNC0638 also increased KDM7A expression in a dose-dependent manner in PANC-1-R cells (Fig. 3E). The binding of G9a to KDM7A promoter was reduced in G9a-depleted cells (Fig. 3F). In addition, two repression markers H3K9 and H3K27 methylation were reduced while the recruitment of MLL and the methylation of H3K4 were increased in the KDM7A promoter (Fig. 3F). These data suggested that KDM7A is a repression target of G9a and the downregulation of KDM7A is involved in the inhibition of E-cadherin by G9a.

### *In vivo* evidence supports the regulation of KDM7A and PCL3 by G9a

**Figure 4:**
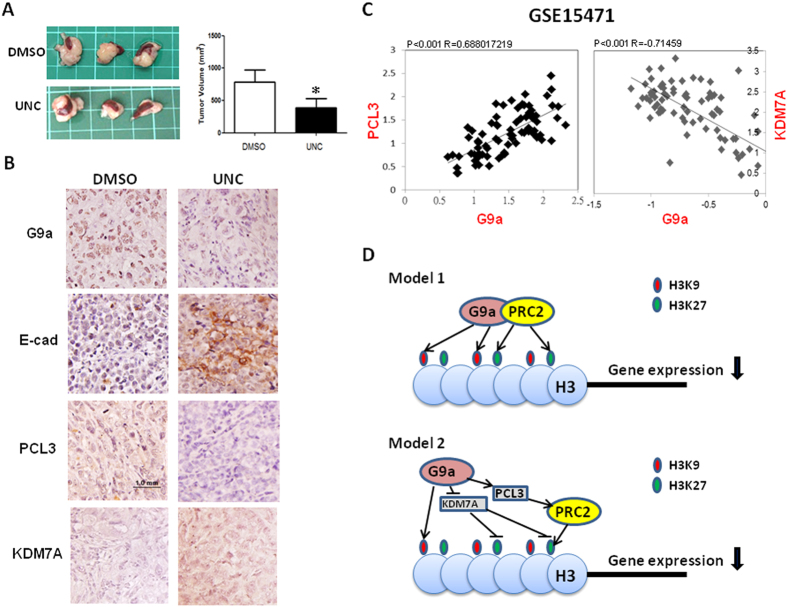
Association of KDM7A, PCL3 and G9a in tumor tissues. (**A**) Effect of UNC0638 on the tumor growth of PANC-1-R cells in the orthotopical animals. The picture showed the tumors harvested from the sacrificed amimals and tumor volume was calculated using the equation: tumor volume = (length × width^2^)/2. (**B**) Immunohistochemical analysis to show the expression of G9a, E-cadherin, PCL3 and KDM7A of tumor tissues obtained from mice received vehicle (DMSO) or UNC0638 treatment. (**C**) Correlations between G9a and PCL3 or KDM7A of 78 human pancreatic tumor tissues were analyzed by using the GSE15471 dataset from Oncomine. Correlation coefficient and *p*-value were shown. (**D**) A cartoon illustrates of the proposed models by which G9a orchestrates PCL3 and KDM7A to promote histone H3K27 methylation.

In order to determine the association between G9a, E-cadherin, PCL3 and KDM7A *in vivo*, animal study and bioinformatics approach were performed. PANC-1-R cells were inoculated into the pancreas of the NOD/CB17 mice. Two weeks after tumor cell injection, mice were grouped to receive vehicle (DMSO) or UNC0638 three times per week for 6 weeks. Treatment of UNC0638 for six weeks inhibited tumor growth by 50% (Fig. 4A) and reduced G9a protein level in the tumors as found in UNC0638-incubated cells (Fig. 4B). E-cadherin and KDM7A were up-regulated while PCL3 was downregulated in the tumors (Fig. 4B). The increase of E-cadherin suggested treatment of UNC0638 reversed the EMT alteration induced by G9a. Next, we analyzed the correlation of these genes in a public dataset (GSE15471, Oncomine). The results showed the expression of G9a was negatively associated with KDM7A while it was positively correlated with PCL3 (Fig. 4C). Taken together, these data supported the results of our cell-based study and suggested that G9a may orchestrate PCL3 and KDM7A to inhibit E-cadherin in pancreatic cancer.

## Discussion

In this study, we reveal a novel mechanism by which G9a orchestrates the dynamic change of various histone modifying enzymes to regulate H3K9 and H3K27 methylation of E-cadherin gene promoter to silence its transcription. Direct physical interaction of G9a and PRC2 at a subset of the promoters of developmental and neuronal regulator genes has been demonstrated recently^12^. Results of the study suggested that G9a and PRC2 form a functional complex to methylate H3K9 and H3K27 simultaneously to maintain silencing of specific target genes (model 1, Fig. 4D). We tested whether G9a could modulate H3K27 methylation via an indirect mechanism. Our data showed that G9a up-regulated PCL3, an accessory protein of the PRC2 complex, to promote PRC2 recruitment to E-cadherin gene promoter to introduce H3K27 methylation. Additionally, G9a decreased KDM7A expression to reduce H3K27 de-methylation. Our results suggest G9a promotes H3K27 methylation by regulating PCL3 and KDM7A (model 2, Fig. 4D).

PRC2 complex is the major H3K27 methyltransferase in modulating chromatin structure and gene silencing. Recent studies highlight the importance of PCL proteins in contributing PRC2-mediated H3K27 trimethylation^14^,^17^. PCL proteins including PLC3 bind to histone H3K36m3 and recruit the PRC2 complex via the Tudor domain to promote the methylation of H3K27. It is worth noting that we demonstrate for the first time that G9a directly binds to human PCL3 promoter to increase its transcription. PCL3 was originally identified as a human homologue of *Drosophila* polycomblike gene^18^. Interestingly, this gene is overexpressed in different human cancers and is associated with cancer invasiveness^18^,^19^,^20^,^21^. Currently, the transcriptional regulation of PCL3 gene in normal and cancer cells is still unclear. Our data suggest G9a as an upstream activator of PCL3.

Besides exploring the role of PCL3, we also identify KDM7A as another critical mediator in G9a-mediated H3K27 methylation. KDM7A has been shown to demethylate histone H3K9m2 and H3K27m2 *in vitro* and *in vivo*^16^,^22^. Recent studies suggest that KDM7A functions as a tumor suppressor by inhibiting tumor growth and angiogenesis^23^,^24^. However, it is largely unknown how KDM7A suppresses tumor progression. Here, we show that KDM7A induces de-methylation of H3K9 and H3K27 of E-cadherin promoter to up-regulate its expression suggesting KDM7A is a negative regulator of EMT. We also find that down-regulation of KDM7A is important for G9a to enhance EMT, migration and invasion.

Collectively, we elucidate the underlying mechanism by which G9a promotes H3K27 methylation and identify PCL3 and KDM7A as direct targets of G9a. This study provides new insights how the interplay between different histone modifying enzymes coordinates different histone markers to fun-tune gene transcription.

## Methods

### Cell lines, reagents and plasmids

Pancreatic cell lines were grown in Dulbecco’s modified Eagles medium containing 10% fetal bovine serum (FBS). RNA interference expression plasmids specific for G9a, KDM7A and PCL3 were purchased from the National RNAi Core Facility (Academia Sinica, Taiwan). The sources of antibodies are: G9a from Epitommics (Burlingame, CA); E-cadherin and EZH2 from BD (San Jose, CA); H3K9m2, H3K9m3, H3K27m2, H3K27m3, H3K4m2 and H3K4m3 were from Cell Signalling (Danvers, MA); KDM7A, MLL1 and SETD1A were from Abcam (Cambridge, MA). PCL3 and FLAG were from Sigma-Aldrich (St. Louis, MO). UNC0638 was purchased from Cayman Chemical (Ann Arbor, MI). KDM7A expression plasmid was kindly provided from Dr. Masabumi Shibuya (Jobu University, Japan).

### Lentivirus production and delivery shRNA

For lentivirus particle production, 293 FT cells were plated in 10-cm dishes (1 × 10^6^ cells per dish) transfected by Lipofectamine 2000 (Invitrogen) according to the manufacture’s protocol. The medium were changed the next day, and virus particles were harvested by collecting the medium at 48 and 72 h post-transfection. The medium was passed through 0.45-μm filter and stored at −80 °C. For lentivirus transduction, PANC-1 cells were infected with media containing virus and polybrene (8 μg/ml). After 24 h, media were replaced with fresh medium containing 2.5 μg/ml of puromycin to select infected cells.

### shRNA

shRNA sequences used are listed in *Supplementary Information*.

### Histone extraction

Cells were harvested and washed twice with cold phosphate-buffered saline (PBS). Cells were re-suspended in Triton extraction buffer (PBS containing 0.5% Triton X-100 (v/v), 2 mM phenylmethylsulfonyl fluoride) for 10 min on ice. The cell pellets were collected by centrifugation at 2000 rpm for 10 min at 4 °C, and were washed by Triton extraction buffer twice. The pellets were extracted in 0.2 N HCl overnight at 4 °C. After that, supernatant containing histone proteins was harvested at 13000 rpm for 10 min and protein concentration was determined using Bradford assay.

### RT-qPCR analysis

Total RNA was extracted and quantitative RT-PCR was performed as described previously^25^ using the primers shown in *Supplementary Information*.

### ChIP-qPCR

ChIP-qPCR assay was performed as described previously^11^ and signals were presented as a ratio of the control. Statistical significance was analyzed using a Student *t* test. Antibodies used for ChIP assay and primers/probes used for qPCR analysis are included in *Supplementary Information*.

### Cell migration and invasion assay

Cell migration and invasion ability were performed as previously described^25^. Briefly, 5 × 10^3^ cells were seeded in 24-well transwell units with polycarbonate filters (pore size 8 μm) coated with 1% gelatin for invasion assay. After removal of the cells in the upper surface of the filters, migrated and invaded cells were fixed and stained with 0.05% Trypan Blue.

### Animals study

Briefly, mice were anesthetized under isoflurane gas. PANC-1-R (1 × 10^6^) cells in 35 μL of HBSS were injected into the neck of pancreas of 7-wk-old C.B17/lcr-scid/scidJcl mice. Two weeks later, the animals were divided into the DMSO- or UNC0638-treated groups. UNC0638 was given at the dose of 5 mg/kg by intraperitoneal injection three times a week. After 6 weeks, mice were sacrificed for further analysis. The tumor volume was calculated using the equation: tumor volume = (length × width^2^)/2. All experiments were performed in accordance with the animal care and use guideline of National Health Research Institutes and the protocol and method were approved by the Animal Care and Use Committee of National Health Research Institutes.

### Immunohistochemistry

Tumor samples were de-paraffined in xylene and endogenous peroxidase activity was blocked with 4% H_2_O_2_. Sections were stained with indicated primary antibodies, appropriate secondary antibodies and developed by the Envission system (Dako, Denmark). Finally, sections were counterstained with hematoxylin and analyzed under a microscope.

### Oncomine data analysis

Oncomine (http://www.oncomine.org) is a cancer microarray database and integrated data-mining platform. Pancreatic cancer data set GSE15471 (sample size = 78) was used to analyze the correlation between G9a and PCL3 or KDM7A expression.

### Statistics

The experiments were repeated three times. Results are expressed as Mean ± S.E. Two-tailed Student’s *t* test was used for intergroup comparisons. *P* value less than 0.05 was considered statistically significant.

## Additional Information

**How to cite this article**: Pan, M.-R. *et al.* G9a orchestrates PCL3 and KDM7A to promote histone H3K27 methylation. *Sci. Rep.* **5**, 18709; doi: 10.1038/srep18709 (2015).

## Supplementary Material

Supplementary Information
