# Supplementary material for: G9a orchestrates PCL3 and KDM7A to promote histone H3K27 methylation
Source: Sci Rep. 2015 Dec 21;5:18709. doi: 10.1038/srep18709 (PMC4685317; doi:10.1038/srep18709)
Supplement: Supplementary Information [file srep18709-s1.pdf]

G9a orchestrates PCL3 and KDM7A to promote histone H3K27 methylation

Mei-Ren Pan[1], Ming-Chuan Hsu[2], Li-Tzong Chen[2,3], Wen-Chun Hung[2,4]

[1]Graduate Institute of Clinical Medicine, College of Medicine, Kaohsiung Medical University, Kaohsiung 804, Taiwan
[2]National Institute of Cancer Research, National Health Research Institutes, Tainan 704, Taiwan
[3]Division of Hematology/Oncology, Department of Internal Medicine, National Cheng Kung University Hospital, College of Medicine, National Cheng Kung University, Tainan 704, Taiwan
[4]Institute of Basic Medicine, College of Medicine, National Cheng Kung University, Tainan 704, Taiwan

Supplementary Information
Supplementary figure legends

Supplementary Figure 1- Proliferation of gemcitabine-resistant PANC-1-R cells is similar to parental PANC-1 pancreatic cancer cells.

Supplementary Figure 2- Increase of G9a, H3K9 and H3K27 methylation in gemcitabine-resistant pancreatic cancer cells.

Supplementary Figure 3- Inhibition of G9a decreased cell migration and invasion without significantly affecting cellular proliferation of PANC-1-R cells.

Supplementary Figure 4- Inhibition of H3K9 and H3K27 methylation by overexpressing methyltransferase-dead G9a (DN-G9a).

Supplementary Figure 5- Inhibition of G9a by shRNA or UNC0638 reduced H3K27 methylation in lung and breast cancer cells.

Supplementary Figure 6- UNC0638 treatment decreased the binding of PCL3 to E-cadherin promoter in PANC-1-R cells.

Supplementary Figure 7- UNC0638 treatment reduced PLC3 expression and decreased the binding of G9a to PCL3 gene prometer in PANC-1-R cells.

Supplementary Figure 8- Specific reduction of PCL3 by UNC0638.

Supplementary Table 1- Primer sequences used in this study.

Supplementary Figure 1- Proliferation of gemcitabine-resistant PANC-1-R cells is similar to parental PANC-1 pancreatic cancer cells. Panc-1 and gemcitabine-resistant Panc-1-R cells were seeded at the density of 2000 cell/per well in 96-well plates. After different times, MTT assay was performed to investigate cellular proliferation. Results were the average of two independent assays.

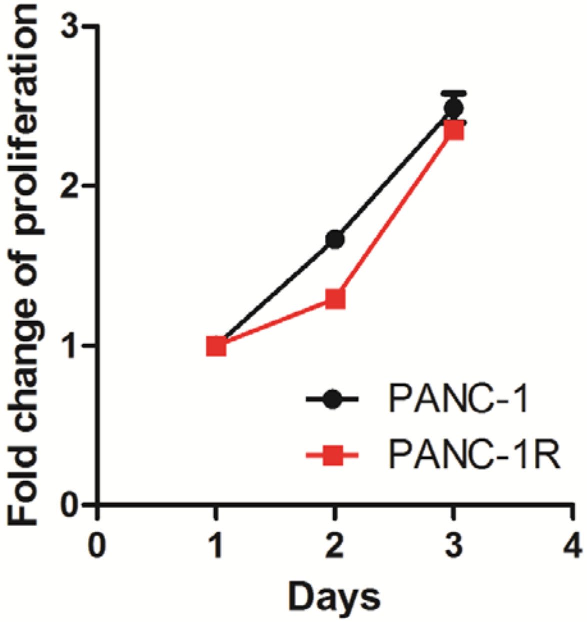

Supplementary Figure 2- Increase of G9a, H3K9 and H3K27 methylation in gemcitabine-resistant pancreatic cancer cells. The protein level of G9a and the methylation status of H3K9 and H3K27 were compared in Panc-1 and gemcitabine-resistant Panc-1-R cells by western blotting.

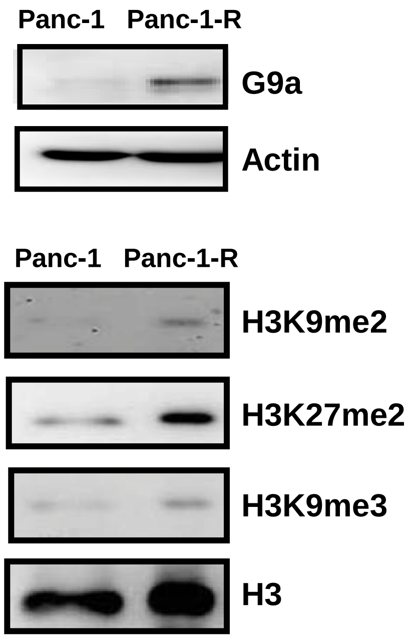

Supplementary Figure 3- Inhibition of G9a decreased cell migration and invasion without significantly affecting cellular proliferation of PANC-1-R cells. (A) Knockdown of G9a (shRNA) or ectopic expression of methyltransferase-dead G9a (DN-G9a) reduced cell migration and invasion of Panc-1-R pancreatic cancer cells. (**B**) The proliferation rate of PANC-1-R cells transfected with control or G9a shRNA.

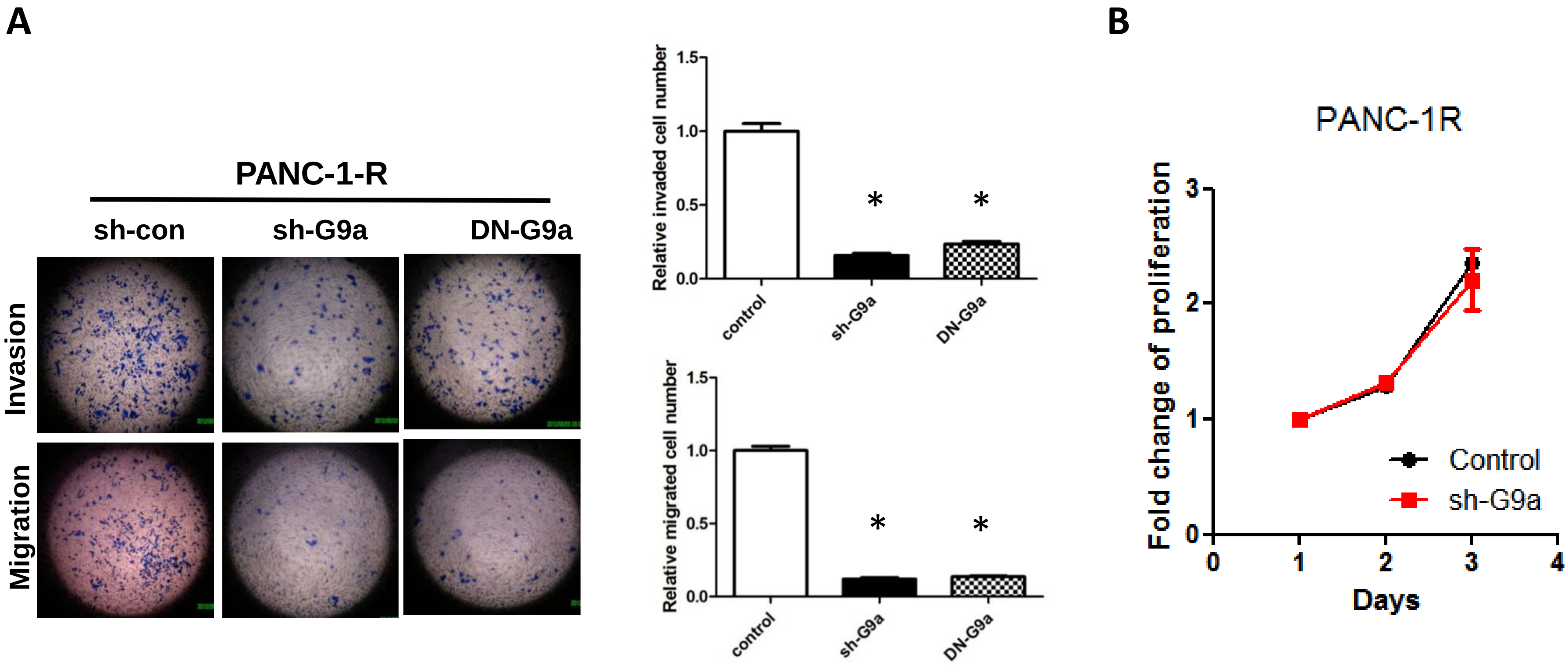

Supplementary Figure 4- Inhibition of H3K9 and H3K27 methylation by overexpressing methyltransferase-dead G9a (DN-G9a). Overexpression of methyltransferase-dead G9a decreased both H3K9 and H3K27 methylation in PANC-1-R cells.

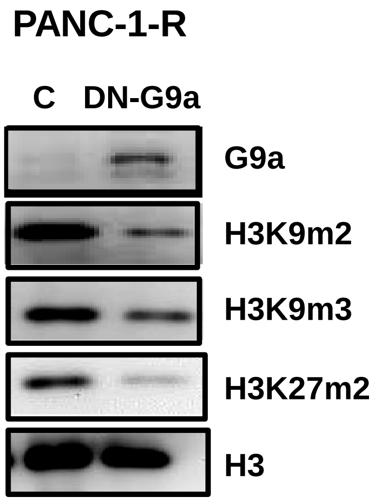

Supplementary Figure 5- Inhibition of G9a by shRNA or UNC0638 reduced H3K27 methylation in lung and breast cancer cells. (A) Knockdown of G9a decreased both H3K9 and H3K27 methylation in A549 lung cancer cells. (B) UNC0638 treatment decreased H3K27 methylation in A549 and MDA-MB-231 cells.

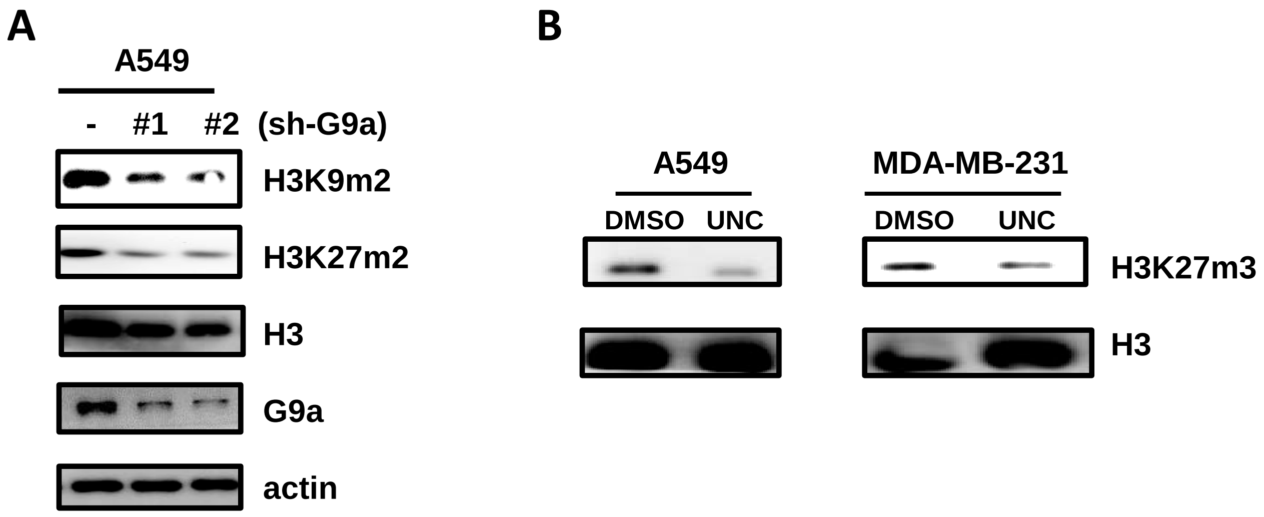

Supplementary Figure 6-UNC0638 treatment decreased the binding of PCL3 to E-cadherin promoter in PANC-1-R cells.

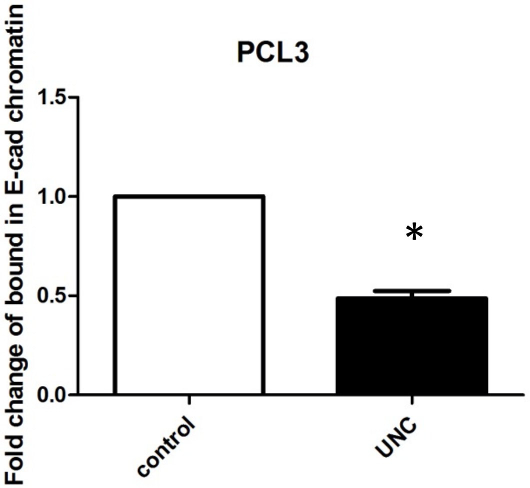

Supplementary Figure 7-UNC0638 treatment reduced PLC3 expression and decreased the binding of G9a to PCL3 gene prometer in PANC-1-R cells.

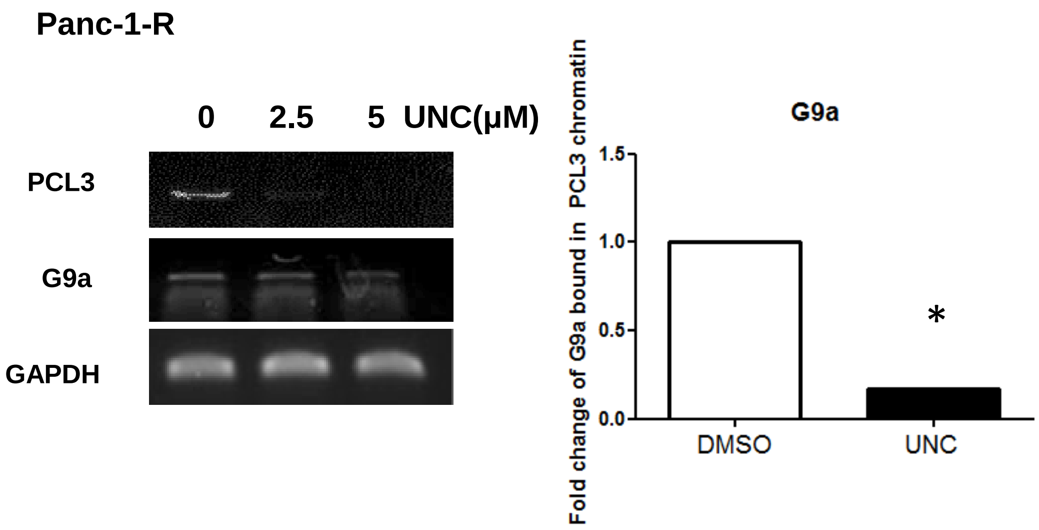

Supplementary Figure 8- Specific reduction of PCL3 by UNC0638. UNC0638 treatment reduced PLC3 protein in a dose-dependent manner. The protein level of EZH2 was decreased slightly. Other components of the PRC2 complex were not affected.

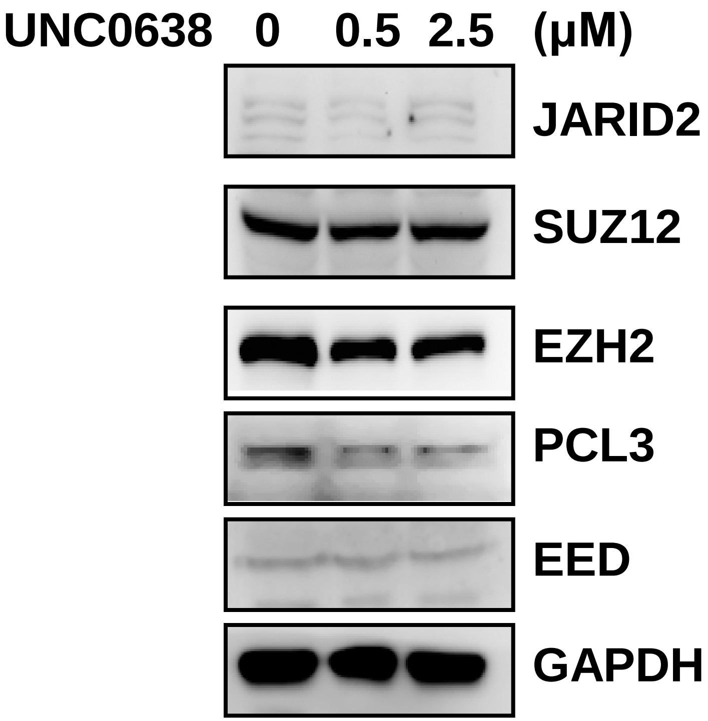

Supplamantary Table 1. Primer sequences used in this study

Assay	Target	Sequence (5'→ 3')	Amplicon (bp)
shRNA	shG9a-C	GCTCCAGGAATTTAACAAGAT	
	sh-G9a-E	CTCCAGGAATTTAACAAGATT	
	sh-KDM7A-C	TGGATTTGATGTCCCTATTAT	
	sh-KDM7a-E	TTAGACCTGGACACCTTATTA	
	sh-PCL3-A	CCTCGTGACTTTCGAAGATAA	
	sh-PCL3-B	CCTGGCTAGCATATTTGACTT	
	sh-PCL3-C	CCCACCTCAAGTCATCTATCA	
	sh-PCL3-D	CAACGCTCTGAACAGTTATAA	
	sh-PCL3-E	ACCACCTGGCTAGCATATTTG	
Real-PCR	E-cadherin	F:CCTGGGACTCCACCTACAGA<br>R: GGATGACACAGCGTGAGAGA	407
	SUZ12	F:GAGCACGTCCAGGCTGACCAC<br>R:TACTGGAAACTGCAAGGGACGGGA	475
	JARID2	F:GGTCCGCGCTCAGGTGGAGA<br>R:AGCTTGCTGCGGAAGCCGTT	594
	PCL3	F:CCAGTATGTGCTGTGCCGGTGG<br>R: CAGGTGTGGCCCTTCTGCTTGG	444
	EED	F:GCCTGCGGCCAAGAAGCAGA<br>R: AGCCAGCAGAGGATGGCTCGT	448
	EZH2	F:GGGACTAGGGAGGTGGAAGA<br>R: GCTGTGCCCTTATCTGGAAA	359
	G9a	F: TGGGAAAGGTGACCTCAGAT<br>R: TCCCTGACTCCTCATCTTCC	336
	KDM7A	F: CGAGTGCGATATCTGCAAGG<br>R: GTCATGTCTGTGCCAGTTCCT	154
	GAPDH	F:GAGTCAACGGATTTGGTCGT<br>R: TGTGGTCATGAGTCCTTCCA	511
Chip primer	E-cadherin promoter (-80~+88)	F: CAGGTGAACCCTCAGCCAATCAGC<br>R: GTGCGGTCGGGTCGGGCC	168
	E-cadherin promoter (-10434~-10236)	F: CCCGCCCCAGCTGTGTCATTTT<br>R: AATGGTGCCCATCCACGTGG	199
	PCL3 promoter (-20~+218)	F:GGTCCCACTTGGAGTCTGG<br>R:AATAATTCGCGGGAAAACG	238
	KDM7A promoter (-1584~-1343)	F:CTCCCTCCCTTTCCTTTCCT<br>R: TGGTGAAACCCCGTCTCTAT	237
